# Potential Antifungal Effects of Calcium Oxide/Zinc Oxide Nanosuspension on Mycelial Growth of *Neoscytalidium dimidiatum* (Ascomycota, Botryosphaeriaceae) Associated With Pistachio Dieback

**DOI:** 10.1111/1758-2229.70144

**Published:** 2025-07-10

**Authors:** Seyedeh Fatemeh Shojaei, Hamid Mohammadi, Nazanin Foroutan, Saleh Panahandeh

**Affiliations:** ^1^ Department of Chemical Engineering, Faculty of Engineering Shiraz University Shiraz Iran; ^2^ Department of Plant Protection, Faculty of Agriculture Shahid Bahonar University of Kerman Kerman Iran; ^3^ College of Pharmacy and Health Science St. John's University New York New York USA

**Keywords:** foliar fertiliser, fungicide, mycelial growth, nanosuspension, *Neoscytalidium*, synergistic effects

## Abstract

The study aimed to synthesise and evaluate the efficacy of calcium oxide (CaO) and zinc oxide (ZnO) nano‐suspensions, in conjunction with adjuvants, on the mycelial growth of *Neoscytalidium dimidiatum*, a notable fungal pathogen impacting pistachio trees in Iran. The study tackles a significant agricultural challenge by exploring eight different treatments, including both nano and pure forms of calcium oxide (CaO) and zinc oxide (ZnO), as well as polyethylene glycol, peracetic acid, and copper oxychloride. The results indicated a notable reduction in mycelial growth, particularly with the zinc oxide nanosuspension, especially when used in combination with peracetic acid, which revealed a synergistic antifungal effect. Further research is necessary to assess the field applications of these treatments for sustainable plant disease management.

## Graphical abstract

This study characterises and investigates the synthesised zinc and calcium oxide nanosuspensions, revealing that zinc oxide nanosuspensions combined with peracetic acid effectively inhibit the mycelial growth of Neoscytalidium dimidiatum, a harmful pathogen affecting pistachio trees.

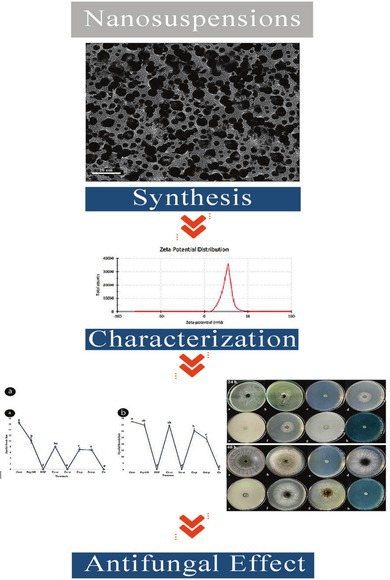

## 1 Introduction

Pistachio ( *Pistacia vera* , Anacardiaceae) is amongst the most widely cultivated nut crops in Iran. According to the Food and Agriculture Organisation (FAO 2018), Iran stands as one of the leading pistachio‐producing countries globally, generating approximately 315,000 metric tons from a cultivation area of 457,000 ha. However, pistachio trees are susceptible to various fungal pathogens that can significantly diminish their productivity. Amongst these pathogens, *Neoscytalidium dimidiatum* has emerged as a significant fungal trunk pathogen, resulting in considerable economic losses in pistachio orchards (Sohrabi 2020; Dervis et al. 2019). Furthermore, the absence of effective long‐term control strategies complicates efforts to mitigate its adverse effects on both tree health and yield (Guney et al. 2022; Nouri, Lawrence, et al. 2018). Members of the *Botryosphaeriaceae* family (Theiss. & P. Syd., Ascomycota, *Botryosphaeriales*) have been identified as endophytes, parasites, and saprophytes on a diverse range of plant species worldwide (Punithalingam 1980; Arx 1987; Crous et al. 2006; Slippers et al. 2007). Most species within this family are recognised as causal agents of various plant diseases, including fruit rots, leaf spots, dieback, branch and trunk cankers, bud necrosis, wood discoloration, decline, and the eventual demise of affected trees (Van Niekerk et al. 2006; Slippers et al. 2007). Previous studies have shown that certain species of Botryosphaeriaceae pose a significant threat to pistachio trees in various countries (Michailides 1991; Ma et al. 2002; Armengol et al. 2008; Inderbitzin et al. 2010; Moral et al. 2010; Chen et al. 2014, 2015), including Iran (Sohrabi 2020). *Neoscytalidium dimidiatum* (synonyms: *Fusicoccum dimidiatum*, *Torula dimidiata*, *Scytalidium dimidiatum*, *Hendersonula toruloidea*) (Crous et al. 2006) is one recognised species within the *Botryosphaeriaceae* family and has been reported as a serious trunk pathogen of pistachio trees (Dervis et al. 2019). More recently, this species has been documented on pistachio trees in Iran (Sohrabi 2020). Geographically, this fungus is distributed across tropical and subtropical countries and is known to infect various plant species globally. Additionally, there are reports of infections in humans (da Silva et al. 2016; Kuan et al. 2023) and animals (Mery Ruíz‐Cendoya et al. 2010) caused by this fungus. In humans, *Neoscytalidium dimidiatum* can lead to skin and nail infections that resemble dermatomycosis and onychomycosis, as well as more severe diseases affecting the brain, sinuses, and lungs, particularly in immunocompromised individuals (Lacaz et al. 1999; Jo et al. 2021). The pathogen's potential to cause both plant and human diseases highlights its adaptability to different environmental conditions. According to available information, there appears to be no standardised effective treatment for systemic skin infection by *N. dimidiatum* (Tosti et al. 2000).

Many studies have been made in vitro to evaluate the efficacy of a range of chemical fungicides against Botryosphaeriaceae spp. on fruit trees (Brown‐Rytlewski and McManus 2000; Amponsah et al. 2012; Pitt et al. 2012; Twizeyimana et al. 2013) including *Botryosphaeria dothidea* on pistachio trees (Ma et al. 2002). Carbendazim is a practical fungicide that is being used against a range of Botryosphaeriaceae species; however, this compound, like many other chemical compounds, can have dangerous effects on human and environmental health (MSDS, WHS Regulations 2021). Despite the availability of chemical fungicides, their prolonged use raises concerns regarding pathogen resistance, environmental contamination, and potential risks to human health (Lamsal et al. 2011; Vitale et al. 2021). Moreover, conventional fungicide formulations frequently exhibit poor solubility and inconsistent bioavailability, which can diminish their effectiveness (Müller and Peters 1998). In light of these challenges, there is a pressing need for alternative, eco‐friendly, and effective antifungal strategies to manage *N. dimidiatum* infections in pistachio orchards. Several fungicides have been studied as antifungal agents for the control of fungal plant pathogens. Preliminary studies have demonstrated antifungal activities of various nanoparticles and inorganic compounds, such as copper (Cioffi et al. 2005) and zinc oxide (Liu et al. 2009). He et al. (2011) reported that zinc oxide nanoparticles (ZnO NPs) significantly inhibit the growth of *Botrytis cinerea* and *Penicillium expansum*. Mosquera‐Sánchez et al. (2020) reported the antifungal effects of zinc oxide nanoparticles (ZnO‐NPs) on *Colletotrichum* sp. Sardar et al. (2021) also reported a similar response of Cu NPs on *Alternaria citri* as a major fungal pathogen of citrus. Yang et al. (2022) confirmed a preventive effect of peracetic acid on *Cladosporium porophorum*, the causal agent of black stain, a post‐harvest disease in Asian pear. Moreover, Panahandeh and Ahmadi (2022) demonstrated how the efficacy of natural pesticides can be enhanced by adjuvants in pistachio orchards. Polyethylene glycol, a practical adjuvant in pesticide formulations, has also been utilised in the nanoencapsulation of various pesticides in recent years (Ebadollahi et al. 2022). The influence of particle size and the dispersibility of certain fungicides on plant diseases have been explored (Yamamoto et al. 2018). Particle size is recognised as a critical property influencing the antifungal and antibacterial activity of nanoparticles, including ZnO (Lipovsky et al. 2011; Applerot et al. 2009). The antimicrobial action of ZnO is attributed to reactions between water and the surface of the nanoparticles. Despite the advantages of nanoparticles in drug delivery systems, many of them exhibit poor aqueous solubility (Jassim and Rajab 2018). Recently, the use of nanosuspensions has emerged as a novel approach to address solubility challenges in drug delivery systems (Chin et al. 2011). A nanosuspension is a liquid containing nanoparticles that must be stabilised by a suitable surfactant or dispersant (Möschwitzer et al. 2004). Studies have shown that nanosuspensions can enhance the solubility and efficacy of treatments (Chan 2011; Wang et al. 2019). Similarly, it has been demonstrated that aqueous suspensions of small particles increase the antimicrobial activity of ZnO (Yamamoto 2001; Zhang et al. 2007). In a study conducted by Ramezani et al. (2014), nanosuspensions prepared using the sonoprecipitation method were compared to powder compounds, revealing that an optimal ratio of dispersed materials could reduce particle size, thereby enhancing dissolution rates and antimicrobial activity. Nanosuspension preparation can be categorised into bottom‐up processes, which involve constructing nanoparticles from molecules (e.g., micro‐precipitation and melt emulsification), and top‐down processes, which reduce larger particles to nanoscale sizes (e.g., high‐pressure homogenisation and milling) (Krishna and Prabhakar 2011). Amongst the available methods for preparing nanosuspensions, milling techniques are particularly advantageous for large‐scale production and cost efficiency (Jamkhande et al. 2019). Suspension stability is a key consideration in the application of these formulations. Factors such as thermodynamic stability and electrostatic stabilisation, assessed by zeta potential measurements, significantly contribute to the stability of dispersed phases in suspensions (Larsson et al. 2012). Whilst nanoparticles have demonstrated antimicrobial activity against various fungal pathogens, their potential application against *N. dimidiatum* has yet to be explored. This study represents the first effort to synthesise and evaluate the antifungal activity of ZnO and CaO nanosuspensions against *N. dimidiatum*. By employing a top‐down milling approach, we aim to develop a stable and scalable nanosuspension formulation with enhanced solubility and bioavailability, providing a novel alternative to traditional fungicides.

### 1.1 Antifungal Activity of Zinc Oxide on Skin Infections

Nanotechnology has introduced the use of nanoparticles, including silver, copper, gold, sulphur, titanium dioxide, and zinc oxide, as innovative treatments for various skin disorders caused by fungi (Rai et al. 2017). Amongst these, zinc oxide nanoparticles (ZnO NPs) have emerged as a promising antifungal agent, particularly in the treatment of fungal dermatophytic infections in cattle. Their unique properties, such as a high surface area and reactivity, enhance their antimicrobial efficacy against a wide range of fungal pathogens. Research has shown that ZnO NPs exhibit significant antifungal activity by disrupting fungal cell membranes and impairing their metabolic functions. This property is particularly beneficial for addressing dermatophytic lesions, which can cause considerable discomfort and health issues in cattle, ultimately affecting their overall productivity (Alghuthaymi et al. 2021). Furthermore, ZnO NPs are recognised for their biocompatibility and safety, making them an attractive option for veterinary applications. The application of ZnO NPs not only offers an effective treatment strategy against dermatophytes but also contributes to reducing reliance on conventional antifungal drugs, thereby minimising the risk of resistance development in fungal species. Overall, the antifungal activity of zinc oxide nanoparticles represents a novel approach to managing dermatophytic infections in livestock, ensuring improved animal health and welfare (Agarwal et al. 2017).

### 1.2 Zinc Oxide Antifungal Activity Mechanism on Plants

Zinc oxide nanoparticles (ZnO NPs) exhibit potent antifungal effects due to their unique physicochemical properties and various mechanisms of action (Sun et al. 2018). One primary reason for their effectiveness is their ability to generate reactive oxygen species (ROS) when exposed to moisture and light. This process induces oxidative stress, resulting in damage to essential cellular components, including lipids, proteins, and DNA within fungal cells. The oxidative damage leads to the disruption of cell membranes and ultimately results in cell death. The high surface area of ZnO NPs facilitates increased interaction with fungal cell membranes, further enhancing their fungicidal efficacy. Notably, their effectiveness against azole‐resistant strains of *Aspergillus flavus* highlights the potential of ZnO NPs as a reliable alternative for combating resistant fungal infections in agricultural settings. Moreover, the high surface area‐to‐volume ratio of ZnO NPs enables extensive interaction with fungal membranes, enhancing their capacity to penetrate and disrupt these structures. ZnO NPs can inhibit the growth and reproduction of various fungal species, including those resistant to conventional antifungal agents, indicating their potential role as alternative therapeutic agents. Additionally, the biocompatibility and low toxicity of ZnO NPs further underscore their promise in both medical and agricultural applications for efficiently controlling fungal infections (Alhazmi and Sharaf 2023).

## 2 Materials and Methods

### 2.1 Fungal Isolates and Culture Conditions

For the evaluation of the inhibitory effects of the treatments on mycelial growth of *N. dimidiatum*, two isolates IRNM235 (GenBank accession numbers: ITS = PQ461110, *tef‐1α* = PQ472408) and IRNM237 (GenBank accession numbers: ITS=PQ461111, *tef‐1α* = PQ472409) were selected. These isolates were previously collected from pistachio orchards in Kerman Province, Iran, identified based on morphological and molecular analyses, and deposited in the culture collection of the Department of Plant Protection at Shahid Bahonar University of Kerman (CSBU), Kerman, Iran (Sohrabi 2020). Both isolates were maintained on Potato Dextrose Agar (PDA; Oxoid, 39 g/L) and incubated at 25°C for 7 days before use in experiments.

### 2.2 Treatments

Eight treatments used in this experiment were calcium oxide WP (Wet Powder), 2 g/L and SC (Suspension Concentrated), 2 g/L, zinc oxide (WP (Wet Powder), 2 g/L and SC (Suspension Concentrate), 2 g/L), polyethylene glycol 400 (L (Liquid)2 g/L), peracetic acid (SL (Soluble Liquid), 2 g/L), copper oxychloride (WP (Wet Powder), 2 g/L), as the positive control, and distilled water as the negative control (Table 1).

**TABLE 1:** Treatments Used in the Study.

Treatment	Formulation	Commercial name	Company	Concentration (g/L)
Zinc oxide	WP (Wettable Powder)	—	Sigma‐Aldrich	2 g/L
Calcium oxide	WP (Wettable Powder)	—	Sigma‐Aldrich	2 g/L
Polyethylene glycol 400 (PEG 400)	LQ (Liquid)	PEG400	Mobtakeran Shimi	2 g/L
Peracetic acid	SL (Soluble Liquid)	Rumba	Fidar Fasl Golkhaneh (FFG Co.)	2 g/L
Zinc oxide	SC (Suspension Concentrate)	LavaLa Organic Liquid Zinc	Fidar Fasl Golkhaneh (FFG Co.)	2 g/L
Calcium oxide	SC (Suspension Concentrate)	LavaLa Organic Liquid Calcium	Fidar Fasl Golkhaneh (FFG Co.)	2 g/L
Copper oxychloride (positive control)	WP (Wettable Powder)	—	Samiran	2 g/L
Distilled water (negative control)	—	—	—	—

Abbreviations: LQ, liquid; SC, suspension concentrate; SL, soluble liquid; WP, wettable powder.

### 2.3 Synthesis of Nanosuspensions

Nanopowders of ZnO (diameter < 100 nm) and CaO (diameter < 160 nm) were used to synthesise the nanosuspensions with a mean diameter of less than 10 nm. To reduce particle size, the nanopowders were initially milled using a Ball Mill (NabTec, BL5, Iran), at room temperature with high energy (200 rpm) for 4 h. Subsequently, wet milling was conducted using a Basket Mill (Pars Centre, 5 L, Iran) with ceramic balls. To optimise the preparation of nanosuspensions for reproducibility and potential scalability, preliminary milling trials were conducted using various milling durations (1, 2, 4, and 6 h). It was observed that particle size decreased with increasing milling time; however, aggregation began to occur beyond 2 h, and further reductions in particle size became minimal after this point. Consequently, a milling duration of 2 h was selected as the optimal time, striking a balance between particle size reduction and stability, with a consistent final size of less than 10 nm and minimal aggregation. Additionally, the wet milling parameters were refined to ensure reproducibility, which included a milling duration of 2 h, a controlled temperature of 25°C ±2°C, a ball‐to‐powder ratio of 1:3 (one part ceramic balls to three parts input materials), a rotation speed of 35 rpm, and a mixture of ceramic balls in equal proportions (50:50 vol.% from 1.5 and 2.5‐mm balls).

To ensure reproducibility and minimise batch‐to‐batch variation, all milling processes were conducted under identical conditions, with stringent control over the milling environment, including ambient humidity and temperature. The quality of the synthesised nanosuspensions was assessed using dynamic light scattering (DLS) measurements and zeta potential analysis to confirm consistent particle size distribution and stability across different batches. Periodic sampling and characterisation were performed to ensure the uniformity of the produced nanosuspensions. These optimised parameters facilitate both reproducibility and potential industrial scalability.

To synthesise 1000 g of zinc oxide nanosuspension, 250 g (25% w/w) of milled zinc oxide powder was dissolved in 225 g (22.5% w/w) deionised water at 25°C ±2°C. Whilst the basket mill (Pars Centre, 5 L, Iran) was rotating at 15 rpm, 225 g (22.5% w/w) Per Acetic Acid and 300 g (30% w/w) Poly Ethylene Glycol 400 (PEG400) were gradually added to the mixture. The ZnO nanosuspension contains ZnO powder (milled), deionised water, PEG 400 as an adjuvant and Peracetic Acid as a dispersant. The concentrations of PEG 400 and Peracetic Acid were optimised based on preliminary trials with varying concentrations, such as 15%, 20%, 25%, and 30% w/w for PEG 400, and 10%, 15%, 20%, and 25% w/w for Peracetic Acid. These trials were conducted to evaluate the effects of concentration on nanoparticle dispersion, aggregation, and stability, helping identify the optimal concentration for both dispersants. To ensure optimal dispersion and prevent aggregation, the basket mill rotation speed was increased to 35 rpm for 90 min. These parameters were chosen based on prior tests, where the effects of different milling times and dispersant concentrations were assessed to determine their impact on particle size and stability. The preparation of calcium oxide nanosuspension followed a similar protocol, with a slight modification: an additional 200 mL of deionised water was added during milling to account for the reaction between CaO and water. The same concentrations of PEG 400 and Peracetic Acid were used as an adjuvant and dispersant, respectively, and were also considered as separate treatments to evaluate their individual fungicidal effects. Polyethylene glycol (PEG) was selected as a dispersing agent due to its ability to prevent nanoparticle agglomeration through steric stabilisation, thereby enhancing the stability of the nanosuspensions (Morsi et al. 2014). Additionally, PEG serves as a wetting agent by lowering surface tension, which facilitates nanoparticle dispersion and prevents sedimentation, ultimately ensuring better formulation stability (Patel et al. 2016; Gupta et al. 2002). This characteristic is essential for maintaining the long‐term stability of nanosuspensions by minimising particle aggregation and ensuring a homogeneous formulation. Peracetic acid was incorporated for its antimicrobial properties and its efficacy in improving the dispersibility of nanoparticles (Pettigrew et al. 2012; Kitis 2004). Furthermore, it contributes to the stability of the nanosuspension by preventing microbial contamination, which can compromise the long‐term integrity of the formulation (Zhang et al. 2019).

### 2.4 Characterisation of the Powders and Nanosuspensions

The size and morphology of calcium oxide (CaO) and zinc oxide (ZnO) powders and nanosuspensions were analysed using Scanning Electron Microscopy (SEM, Cambridge S360, England) and Transmission Electron Microscopy (TEM, Hitachi HF3300, Japan). The resulting images were processed using the ImageJ programme, allowing for the selection of random particles at different magnifications. The mean diameter was then calculated based on the assumption of a perfect spherical shape. Subsequently, the particle size distribution curve and morphological characteristics of the samples post‐milling were discussed. The particle size distribution, as indicated by the polydispersity index (PDI), mean hydrodynamic diameter, and *Z*‐average, was determined using a Dynamic Light Scattering (DLS) analyser (Malvern, Nano ZS (red badge), ZEN3600, England). This instrument also characterised the thermodynamic stability of the nanosuspensions by measuring zeta potential. In addition to the initial measurements, the zeta potential of both CaO and ZnO nanosuspensions was monitored over time to assess long‐term colloidal stability. Samples were stored at room temperature (approximately 25°C) in tightly sealed glass vials, with zeta potential measurements repeated after 10 and 30 days of storage. The analysis of particle size distribution and zeta potential figures provided insights into the morphology and size distribution of the nanosuspensions. Moreover, contact angle analysis (IFT‐CA, CA‐ES20, Iran) was performed to assess the hydrophilic or hydrophobic characteristics of the samples, with the contact angle between water and dried nanosuspension measured three times for each sample.

### 2.5 Effect of Treatments on *N. dimidiatum*

The eight different treatments (Table 1) were tested with a unit concentration (2 g/L) to determine the fungicidal effects on mycelial growth of *N. dimidiatum* isolates in vitro conditions. The final concentration of the treatments after amending with PDA was 2000 ppm. Each treatment was suspended in sterile distilled water and added to 50°C molten potato dextrose agar (PDA; Oxoid,39 g/L). The amended media was thoroughly mixed, and 25 mL of the solution was poured into sterile Petri dishes (10 cm in diameter) under aseptic conditions. Copper oxychloride and distilled water were used as positive and negative treatments, respectively. Both isolates of *N. dimidiatum* were grown on PDA and incubated at 25°C for 5 days. A colonised agar plug (5 mm diam.) was taken from the margin of actively growing colonies and placed in the centre of Petri dishes containing PDA amended with above‐mentioned treatments. The experiments were repeated twice over 10 days and all inoculated Petri dishes were incubated at 25°C ±1°C in a completely randomised design (CRD) with four replications for each combination of treatment concentration and fungal isolate. After 24 and 48 h the radial growth of fungal isolates was measured for each treatment, and the percentage of mycelial growth inhibition (MGI) was calculated by using the formula presented by da Silva et al. (2015).

$$\mathrm{MGI}\;\left(\%\right)=\left[\left(\mathrm{DC}–\mathrm{DT}\right)/\mathrm{DT}\right]\times 100$$

where MGI% is the mycelial growth inhibition percentage; DC is the mean diameter of fungus colonies in PDA medium without fungicide (control); DT is the mean diameter of fungus colonies in a PDA medium with fungicides (treatments).

### 2.6 Statistical Analysis

All statistical analyses were conducted using SPSS software (Version 25) and MSTAT‐C. The experiment was arranged in a factorial layout within a completely randomised design (CRD). Data were analysed using one‐way analysis of variance (ANOVA), with statistical significance set at *p* < 0.05. Post‐hoc comparisons were performed using Duncan's Multiple Range Test (DMRT) at a significance level of *p* < 0.01 to determine significant differences between treatment means (Gomez and Gomez 1984). DMRT was selected because it controls the Type I error rate across multiple comparisons by ranking means into statistically distinct groups based on the Studentised range statistic (*Q*‐value).

## 3 Results

### 3.1 Powders and Nanosuspensions Characterisation

**FIGURE 1:**
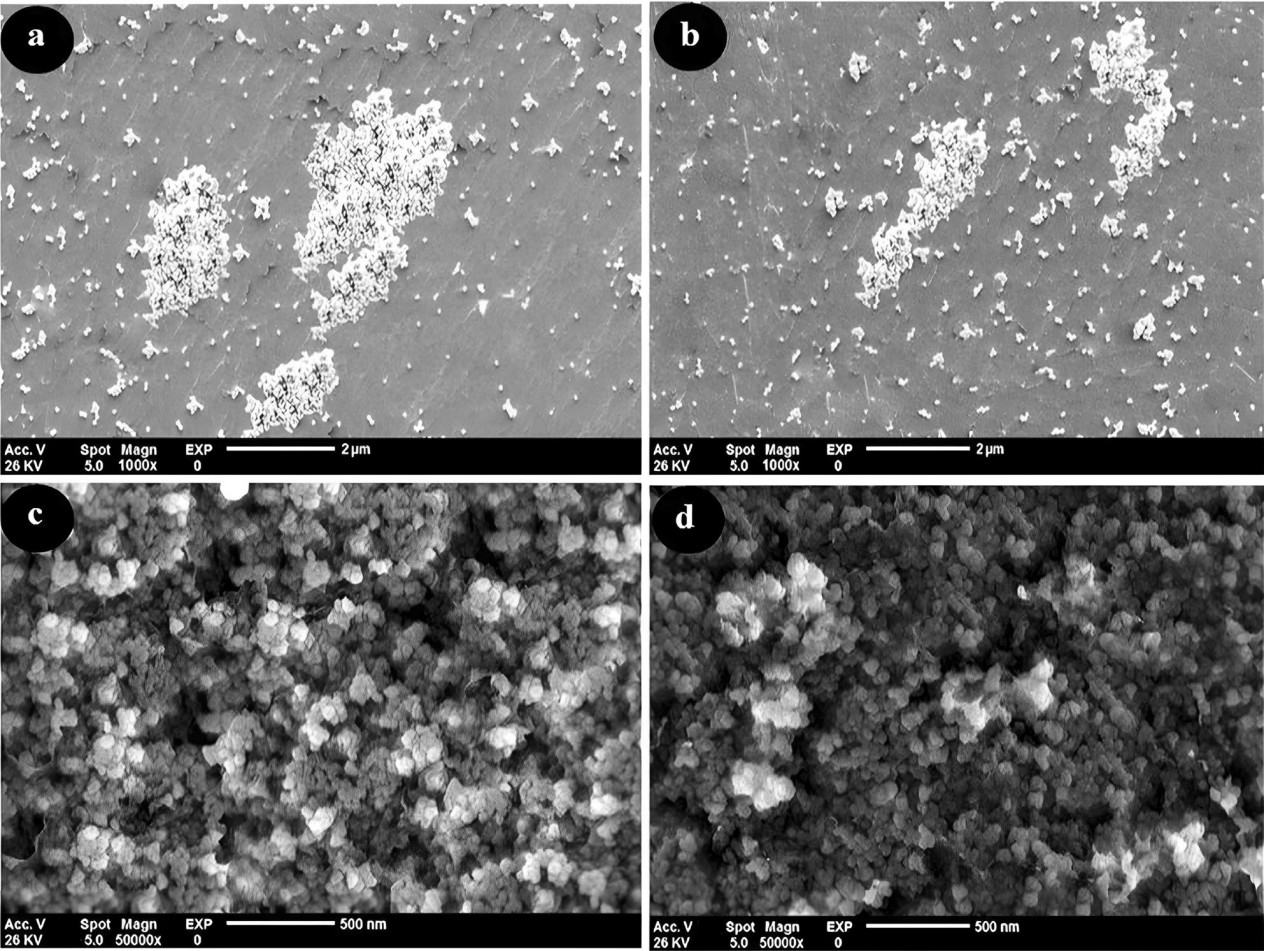
Scanning electron microscopy (SEM) images of (a) CaO powder, (b) ZnO powder, (c) CaO nanosuspension, and (d) ZnO nanosuspension. Magnifications: (a, b) × 1000; (c, d) × 50,000. Scale bars: (a, b) = 2 μm; (c, d) = 500 nm.

**FIGURE 2:**
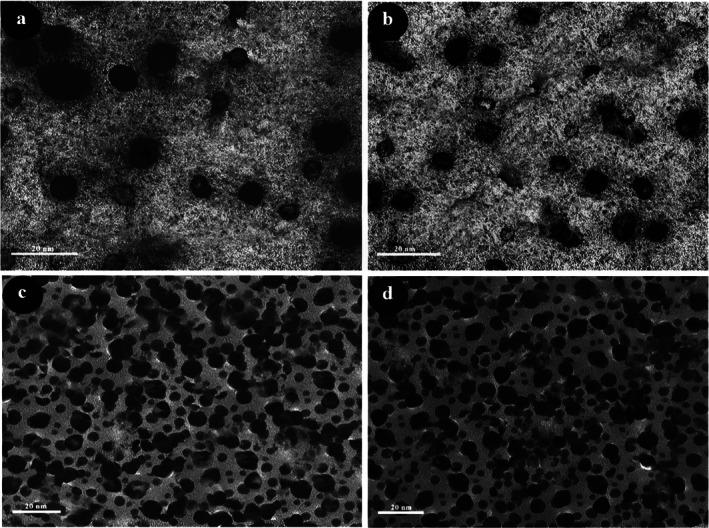
Transmission electron microscopy (TEM) images of (a) CaO powder, (b) ZnO powder, (c) CaO nanosuspension, and (d) ZnO nanosuspension. Scale bars represent 20 nm.

**FIGURE 3:**
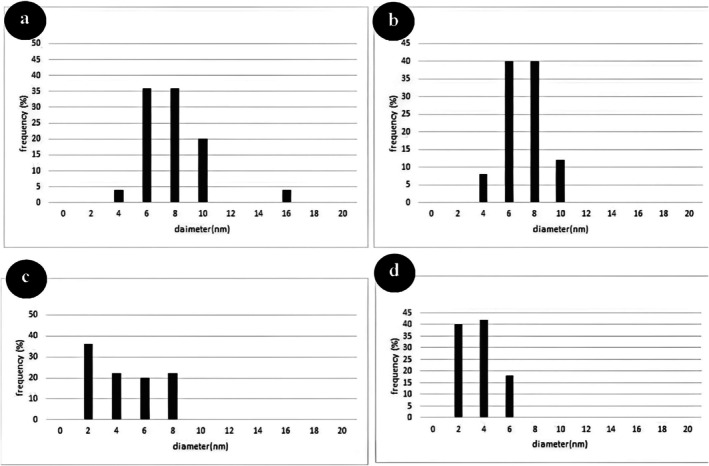
Particle size distribution based on Transmission Electron Microscopy (TEM) analysis for (a) CaO powder, (b) ZnO powder, (c) CaO nanosuspension, and (d) ZnO nanosuspension. Graphs show the frequency (%) of particles as a function of particle diameter (nm).

**FIGURE 4:**
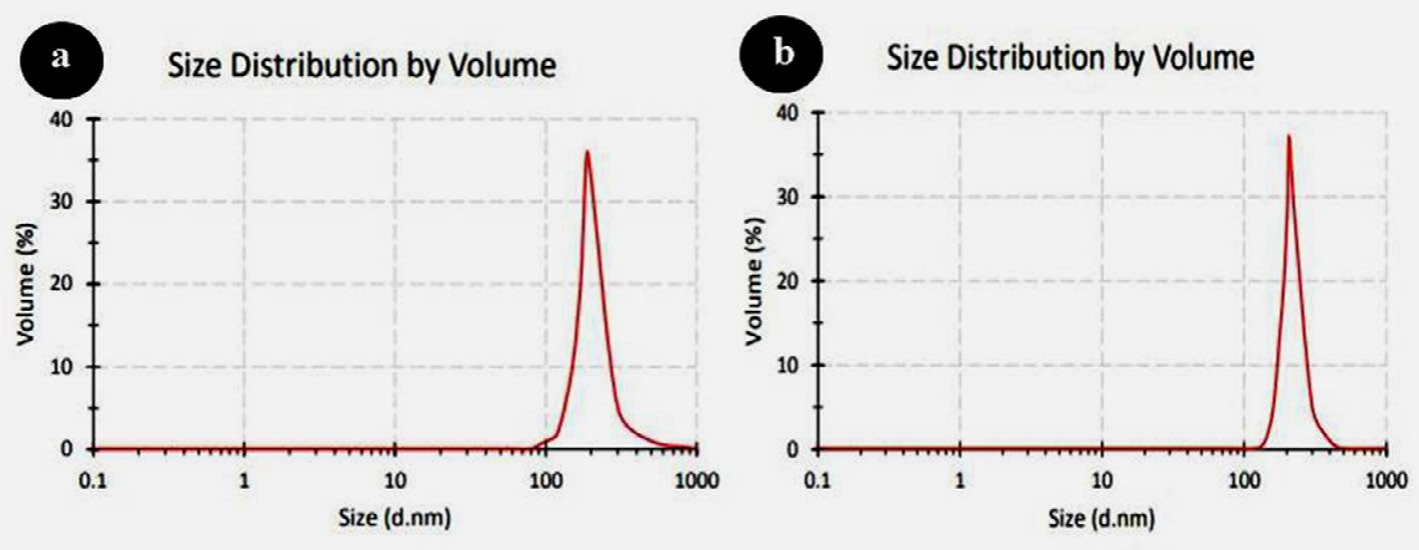
Particle size distribution analysis based on dynamic light scattering (DLS) for (a) CaO nanosuspension and (b) ZnO nanosuspension. The *x*‐axis represents particle diameter (nm), and the *y*‐axis indicates the volume percentage of particles.

**FIGURE 5:**
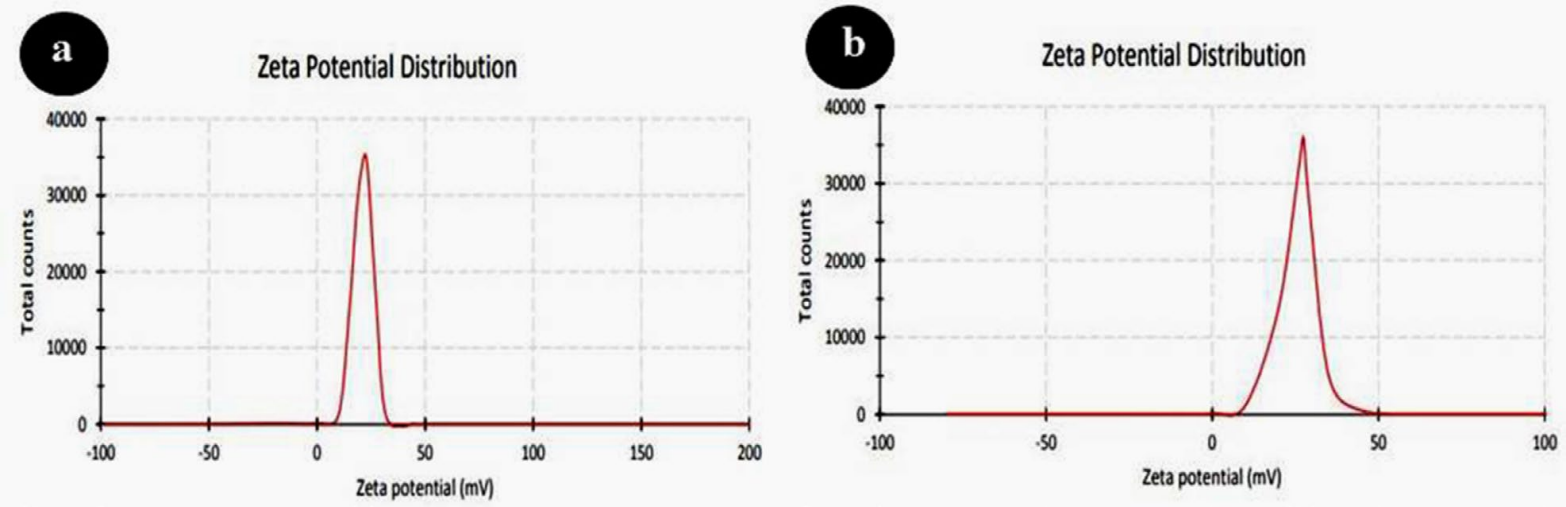
Zeta potential distribution curves for (a) CaO nanosuspension and (b) ZnO nanosuspension. The *x*‐axis represents the zeta potential (mV), whilst the *y*‐axis shows the frequency (%) of particles.

**FIGURE 6:**
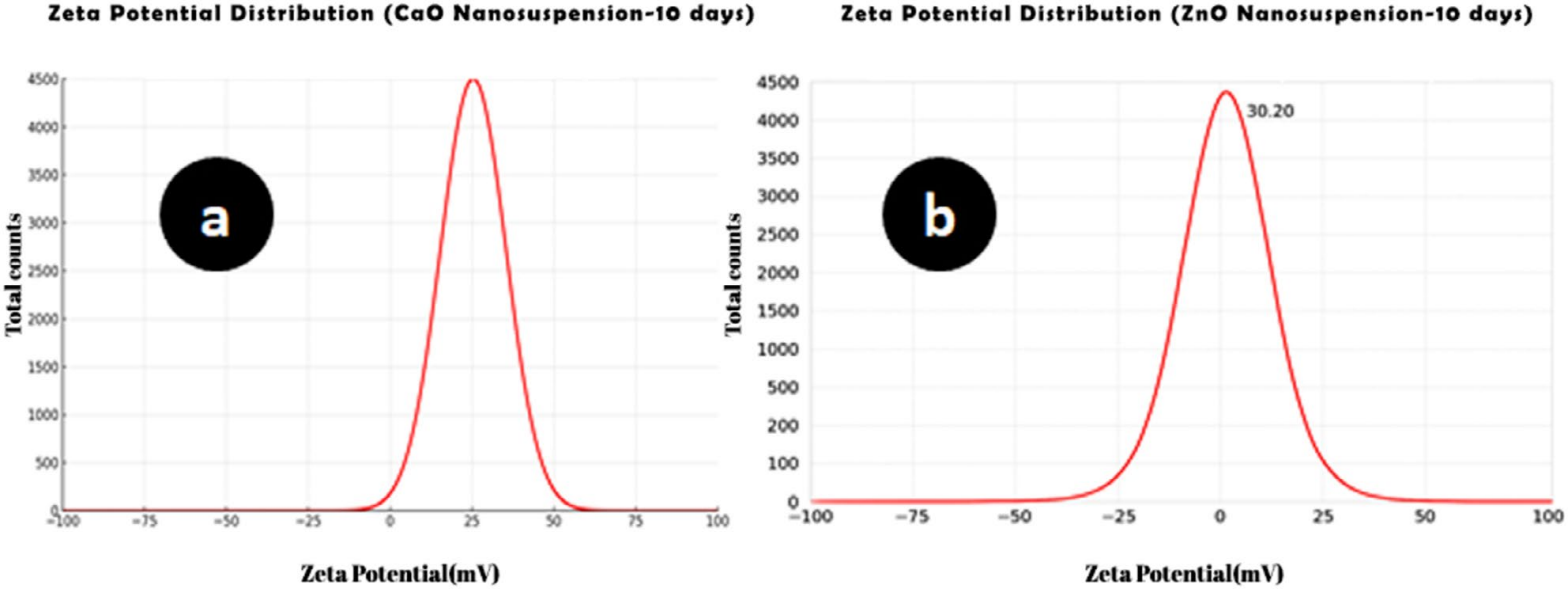
Zeta potential distribution curves after 10 days for (a) CaO nanosuspension and (b) ZnO nanosuspension. The *x*‐axis represents the zeta potential (mV), whilst the *y*‐axis shows the frequency (%) of particles.

**FIGURE 7:**
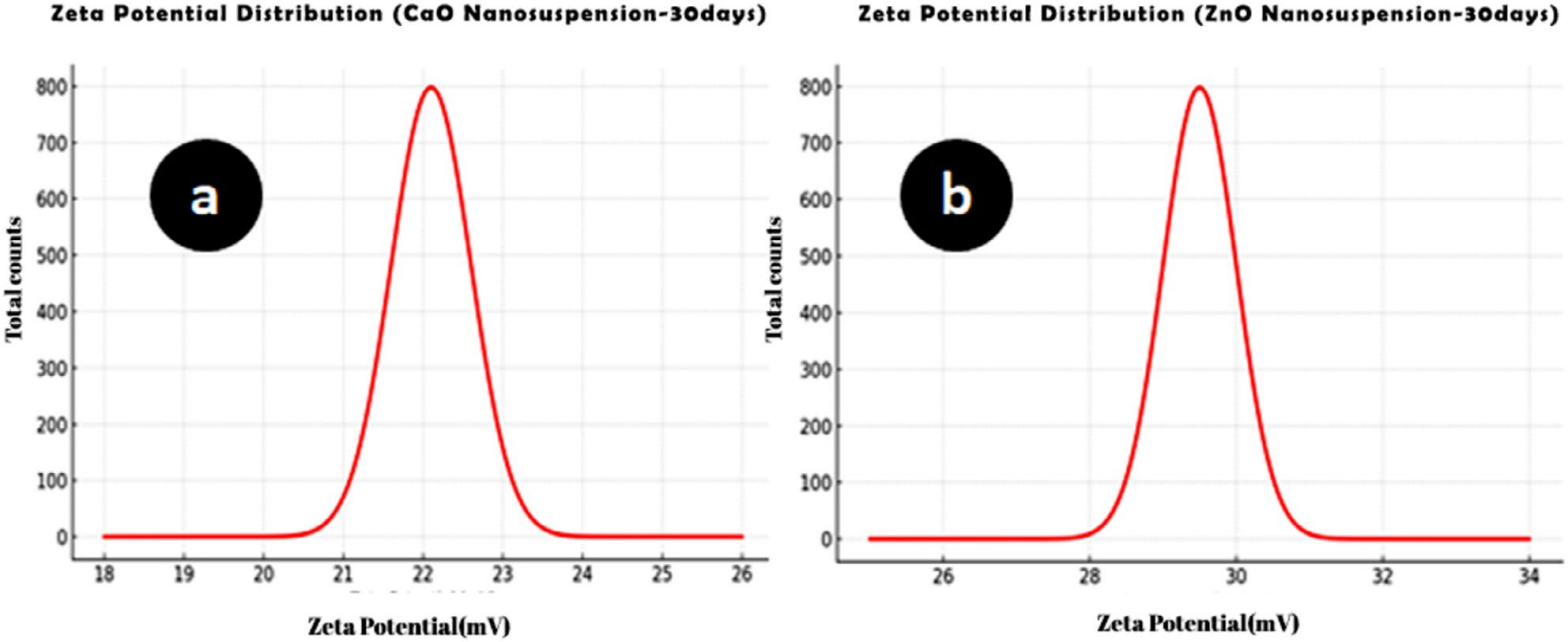
Zeta potential distribution curves after 30 days for (a) CaO nanosuspension and (b) ZnO nanosuspension. The *x*‐axis represents the zeta potential (mV), whilst the *y*‐axis shows the frequency (%) of particles.

The morphology and mean diameter of powders and dried nanosuspensions were investigated by TEM and SEM microscopes with different magnifications (Figures 1 and 2). The results have proved the almost spherical shape of particles in nature. The mean diameter of CaO and ZnO powders based on TEM analysis was 8.86 and 8.17 nm, respectively. This range of size for powders is related to dry milling under optimised situations before nanosuspension synthesis. The mean diameter of CaO and ZnO nanosuspensions was 5.43 and 4.66 nm, which revealed 38.7% and 43.0% reduction of size for CaO and ZnO samples after wet milling, respectively. According to the smaller mean deviation amount (Table 2) and narrow variation in the size distribution curve (Figure 3), it can be concluded that the ZnO nanosuspension sample had better morphology than other samples. In order to investigate the dispersion of nanoparticles in suspensions, hydrodynamic diameter based on volume, *Z*‐average, and polydispersity index (PDI) were discussed with a DLS analyser (Figure 4). The PDI values for ZnO and CaO nanosuspensions were approximately the same (CaO = 0.447, ZnO = 0.441). The zeta potential was measured to study nanosuspensions stability. The zeta potential values for CaO and ZnO nanosuspensions were recorded at 29.61 and 30.83 (mV), respectively. The results have proved that the zeta potential approximation for both samples is sufficient (more than ±30 mV) to be classified as a stable nanosuspension. The zeta potential distribution curve showed a narrow width, which can validate the fine stability of nanosuspension samples (Figure 5). To further investigate the colloidal stability over time, the zeta potential of both nanosuspensions was re‐measured after 10 and 30 days of storage at room temperature (Figures 6 and 7). The ZnO nanosuspension exhibited zeta potential values of 30.20 mV on day 10 and 29.50 mV on day 30, remaining close to the original value of 30.83 mV. This consistency indicates minimal aggregation and sustained electrostatic repulsion, which are characteristics of stable dispersions (Smith et al. 2020; Liu et al. 2017). In contrast, the CaO nanosuspension demonstrated a more pronounced decline in zeta potential, with values dropping to 25.40 mV and 22.10 mV on days 10 and 30, respectively. This reduction suggests a decrease in surface charge and an increase in particle interactions over time. These results highlight that the ZnO nanosuspension exhibits superior long‐term electrostatic stability compared to the CaO nanosuspension. The contact angle was measured three times for each sample with a mean value of 50.68° for CaO and 34.09° for ZnO nanosuspensions. These results indicate that both samples are hydrophilic. However, the higher contact angle observed for the CaO nanosuspension indicates lower wettability compared to that of the ZnO nanosuspension. A higher contact angle is typically associated with lower surface energy and reduced spreading ability, which can adversely affect nanoparticle dispersion and bioavailability in biological systems (Karunakaran, Suriyaprabha, et al. 2015). This lower wettability may account for the diminished antifungal efficacy of CaO nanosuspensions observed in vitro. The notable difference in the mean diameter values obtained from DLS and TEM analyses can be attributed to the measurement methods. In the DLS test, the hydrodynamic diameter of the particles in suspension is measured, which is larger due to the presence of dispersants and adjuvants. In contrast, the diameter assessed by TEM reflects the actual size of dried particles, leading to smaller measurements compared to those obtained via DLS.

**TABLE 2:** Nanosuspension characterisation summary.

Parameter	CaO‐P	ZnO‐P	CaO‐Sc	ZnO‐Sc
Mean diameter (nm)	8.86 ± 2.27	8.17 ± 1.38	5.43 ± 2.18	4.65 ± 1.33
*Z*‐Average (nm)	—	—	199.2	193.5
Mean hydrodynamic diameter (nm)	—	—	204	189
PDI	—	—	0.447	0.441
Zeta Potential (mV)	—	—	29.61	30.83
Zeta potential after 10 days (mV)	—	—	25.40	30.20
Zeta potential after 30 days (mV)	—	—	22.10	29.50
Mean contact angle (°)	—	—	50.68	33.99

Abbreviations: °, degrees; mV, millivolts; nm, nanometres; *P*, powder; PDI, polydispersity index; Sc, suspension concentrate.

### 3.2 Effect of Treatments on Mycelial Growth of *N. dimidiatum* Isolates

#### 3.2.1 Isolate IRNM235

**FIGURE 8:**
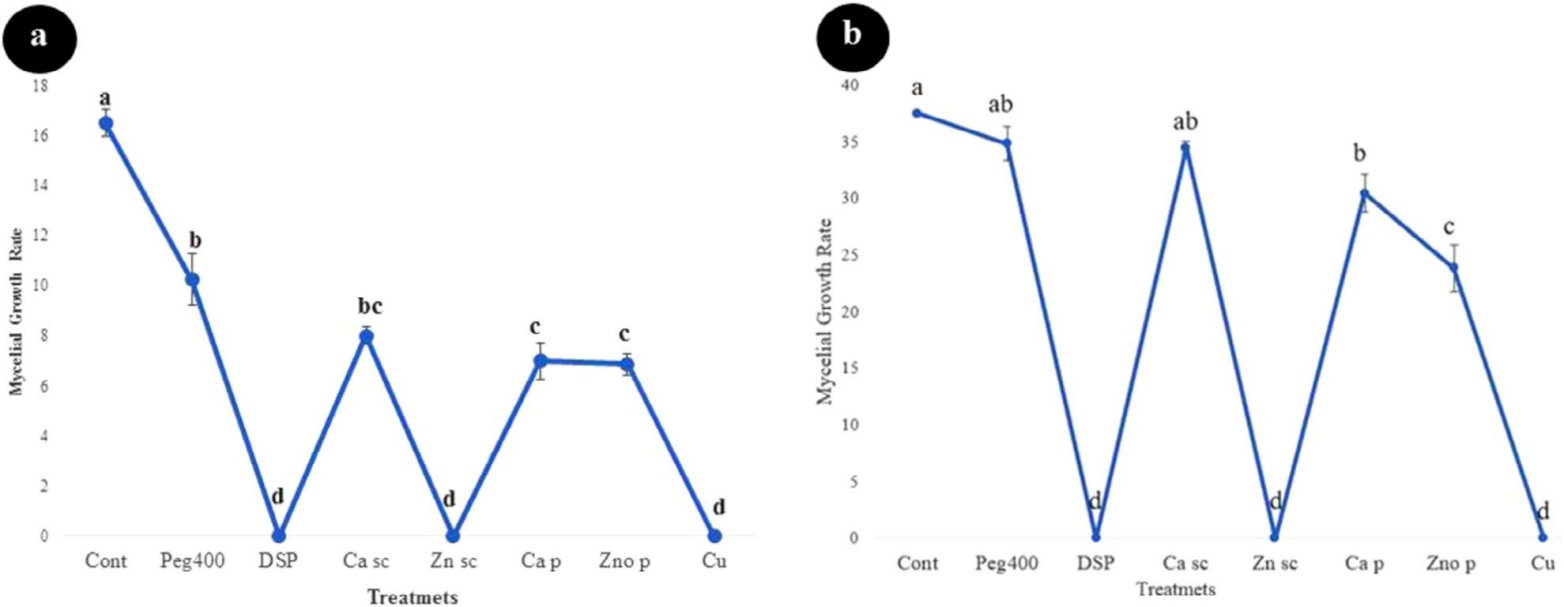
Fungicidal effects of different treatments on the mycelial growth of *Neoscytalidium dimidiatum* (IRNM235 isolate) after (a) 24 h and (b) 48 h. Treatments include Control (Cont), Polyethylene Glycol 400 (PEG400), Peracetic Acid (DSP), Calcium oxide nanosuspension (Ca sc), Zinc oxide nano suspension (Zn sc), Calcium oxide powder (Ca p), Zinc oxide powder (ZnO p), and Copper oxychloride (Cu). Different letters above the bars indicate statistically significant differences amongst treatments (*p* < 0.05).

Based on our findings, the highest mycelial growth rate of IRNM235 isolate was recorded in control (mean: 16.20 mm) followed by Peg400 (mean: 10.00 mm) treatment, calcium oxide suspension (mean: 8.00 mm), calcium oxide powder (mean: 7.00 mm), and zinc oxide powder (mean: 6.87 mm) (*F* = 185, dft,e = 7,8, *p* ≤ 0.001). The results of different treatments on mycelial growth of *N. dimidiatum* (isolate IRNM235) after 24 and 48 h are shown in Figure 8a,b. According to Figure 8a, all treatments significantly reduced the mycelial growth of IRNM235 isolate compared to the control after 24 h incubation. However, three of the eight treatments tested—peracetic acid (DSP), zinc oxide suspension (SC), and copper oxychloride—exhibited significantly greater inhibitory effects compared to the other treatments. No growth was observed in the Petri plates containing these treatments, resulting in no significant differences in mycelial inhibition data amongst them. Similarly, there was also no significant difference in mycelial inhibition data amongst three other treatments: the pure powders of calcium, zinc oxide, and calcium suspension.

**FIGURE 9:**
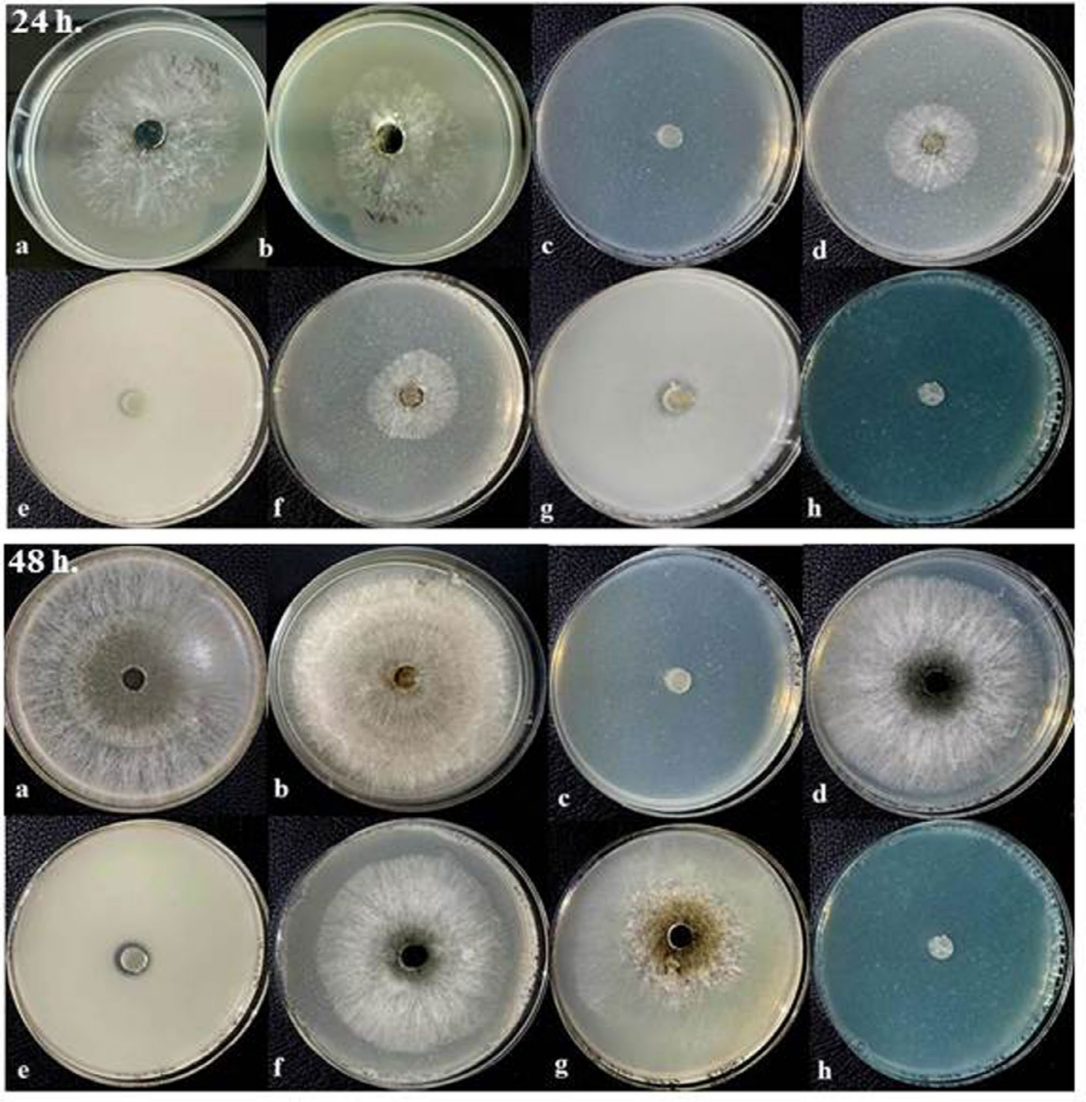
Representative images of *Neoscytalidium dimidiatum* (IRNM235 isolate) mycelial growth under different treatments after 24 h (upper panel) and 48 h (lower panel). Treatments: (a) Control, (b) PEG400, (c) Peracetic Acid, (d) Calcium oxide nanosuspension, (e) Zinc oxide nanosuspension, (f) Calcium Oxide (powder), (g) Zinc Oxide (powder), (h) Copper Oxychloride.

Relatively similar results were also observed after 48 h for this isolate Figure 8b. In this test, only five treatments significantly reduced the mycelial growth of the IRNM235 isolate compared to the control. Again, peracetic acid, zinc oxide suspension, and copper oxychloride had the greatest effect in inhibiting the mycelial growth of this fungus. These treatments were highly effective at inhibiting the mycelial growth of the IRNM235 isolate, and no growth occurred in Petri plates including these treatments. Therefore, these treatments gave complete growth inhibition of this fungus isolate. Whereas, at the same time, control Petri plates had been covered by fungal mycelium. A maximum mycelial growth of 8.00 mm was recorded in the control treatments, followed by Peg400 (mean: 34.87 mm), calcium suspension (mean: 34.50 mm), and zinc oxide (mean: 23.87 mm). In this regard, three treatments (Peg400, calcium oxide suspension, and calcium oxide powder) did not depict considerable differences compared to the control (*F* = 127.97, dft,e = 7,8, *p* ≤ 0.0001). The results are visually evident in Figure 9.

#### 3.2.2 Isolate IRNM237

**FIGURE 10:**
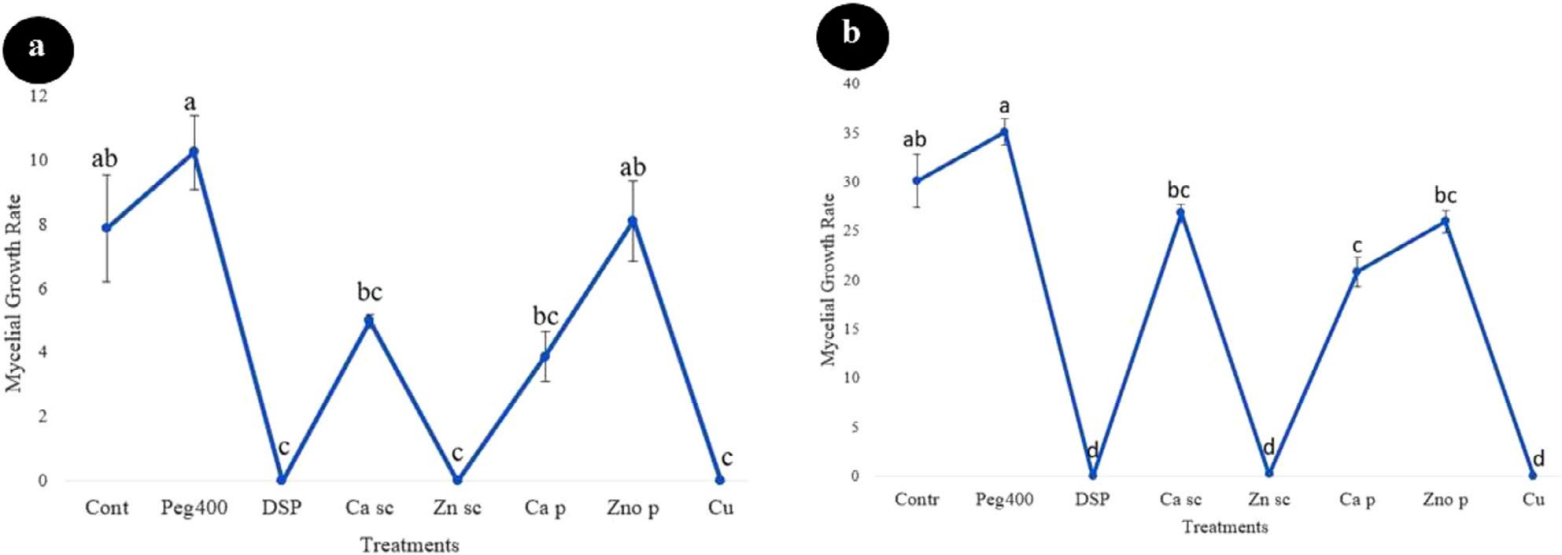
Fungicidal effects of different treatments on the mycelial growth of *Neoscytalidium dimidiatum* (RNM237 isolate) after (a) 24 h and (b) 48 h. Treatments include Control (Cont), Polyethylene Glycol 400 (PEG400), Peracetic Acid (DSP), Calcium oxide nanosuspension (Ca sc), Zinc oxide nanosuspension (Zn sc), Calcium oxide powder (Ca p), Zinc oxide powder (ZnO p), and Copper oxychloride (Cu). Different letters above the bars indicate statistically significant differences amongst treatments (*p* < 0.05).

**FIGURE 11:**
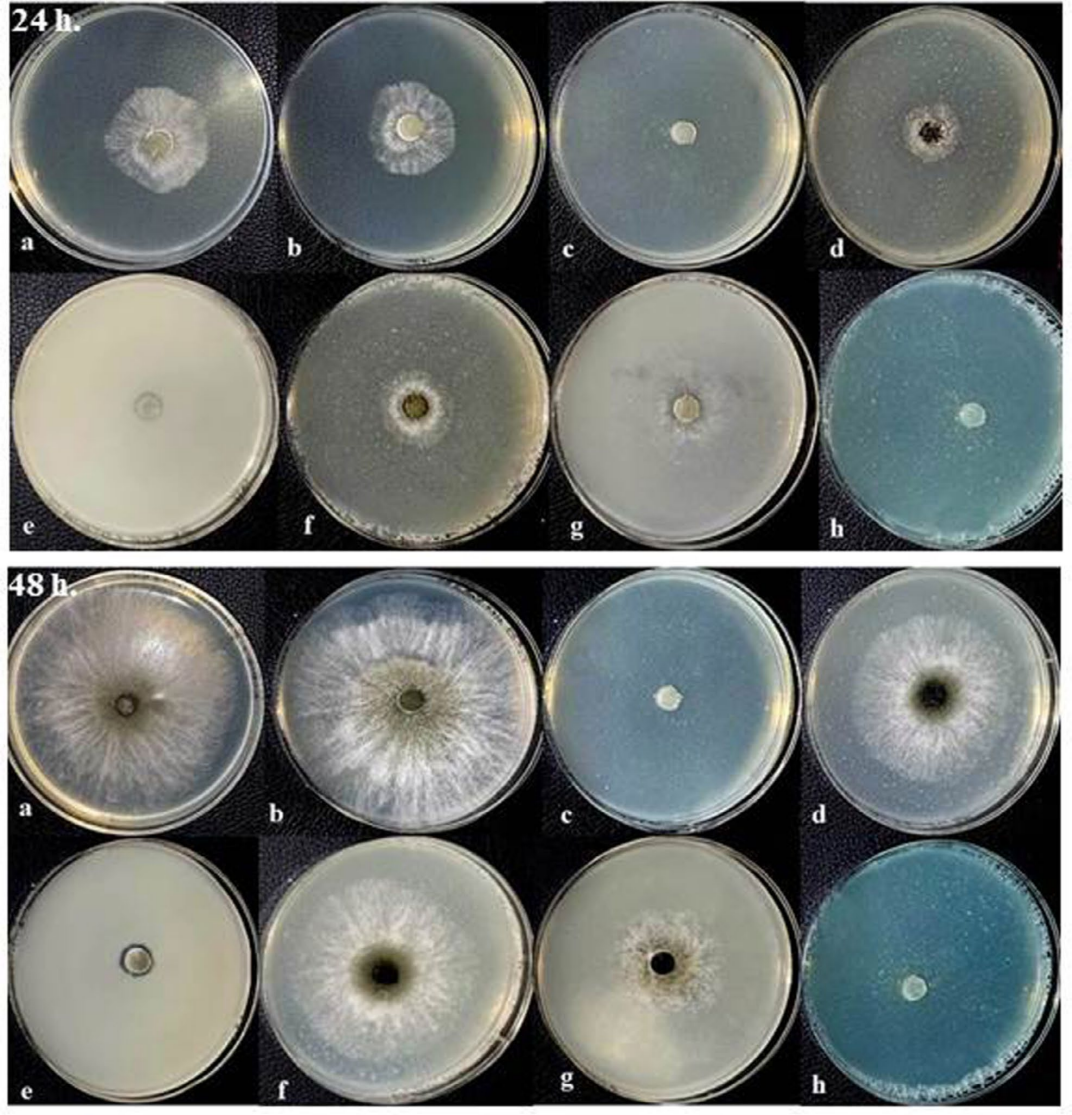
Representative images of *Neoscytalidium dimidiatum* (RNM237 isolate) mycelial growth under different treatments after 24 h (upper panel) and 48 h (lower panel). Treatments: (a) Control, (b) PEG400, (c) Peracetic Acid, (d) Calcium oxide nanosuspension, (e) Zinc oxide nanosuspension, (f) Calcium Oxide (powder), (g) Zinc Oxide (powder), (h) Copper Oxychloride.

Similar results were also obtained on the effect of the tested treatments on the second isolate (isolate IRNM237) of *N. dimidiatum* (Figures 10 and 11). From amongst the tested treatments, only three treatments that were mentioned earlier, viz., peracetic acid, zinc oxide (Sc) and copper oxychloride were found most effective against the mycelial growth of the IRNM237 isolate and showed a significant difference compared to the other treatments as well as the control after 24 h. In this regard, no significant difference was found amongst these three treatments, and all of them completely inhibited the mycelial growth of the isolate. Both treatments of calcium oxide (powder and suspension treatments) reduced the mycelial growth of the IRNM237 isolate (mean: 3.20 and 5.00 mm, respectively) compared to the control (mean: 7.87 mm) (*F* = 21.37, dft,e = 7,8, *p* ≤ 0.001) however, there was no significant difference compared to the control as well as zinc oxide (pure) treatment (mean: 8.12 mm).

The results obtained from the evaluation of treatments on the mycelial growth of the IRNM237 isolate after 48 h indicated that four treatments significantly reduced the mycelial growth of this fungus. Consistent with previous tests, copper oxychloride (Wp), zinc oxide suspension (Sc), and peracetic acid dispersant exhibited the most pronounced inhibitory effects on the mycelial growth of the tested fungus, demonstrating significant differences compared to other treatments. Notably, there were no significant differences amongst these three treatments, all of which were effective in controlling fungal mycelial growth. Although the treatments of calcium suspension (mean: 26.87 mm) and pure zinc oxide (mean: 26.00 mm) did not show significant differences compared to the aforementioned antifungals, pure calcium oxide also demonstrated a notable effect on mycelial growth, with a mean of 20.87 mm, compared to the control group, which had a mean growth of 30.12 mm. Additionally, this study revealed that PEG 400, used as a wetting agent in the suspension concentrate, did not influence *N. dimidiatum* mycelial growth, recording a mean of 34 mm. In contrast, peracetic acid displayed a satisfactory effect in inhibiting the mycelial growth of the fungus in vitro (mean: 0.00). Figure 10b (*F* = 132.16, dft,e = 7,8, *p* = 0.000).

## 4 Discussion

Nanosuspensions hold significant potential for providing stable formulations that are more efficient for their intended applications. The primary objective of nanosuspensions is to preserve the properties of active substances and prevent their degradation (Gulsun et al. 2018). Recent studies have reported enhancements in two critical characteristics—toxicity and stability of pesticides—through the formulation of nanoparticles (Wang et al. 2021; Ebadollahi et al. 2022). The current study represents the first report on the effects of calcium and zinc oxide nanosuspensions synthesised on the mycelial growth rate of *Neoscytalidium dimidiatum* in vitro. We employed a combination of dry and wet milling methods under optimised parameters to achieve uniformly stable nanosuspensions with a mean diameter of less than 10 nm. Previous research has indicated that modifying certain parameters of zinc oxide nanoparticles can enhance their antimicrobial activity (Talebian et al. 2013; Stanković et al. 2013). Size reduction and chemical surface modifications using suitable dopants have been shown to increase the antimicrobial efficacy of various compounds (Kairyte et al. 2013). In our investigation, we modified both effective parameters, successfully reducing the particle size to less than 10 nm and employing peracetic acid for surface modification, to enhance the antimicrobial activity of the nanosuspensions. Our results indicated that these adjustments significantly increased the efficacy of the zinc oxide treatment. Additionally, we evaluated the characteristics of the nanosuspensions, focusing on particle morphology, dispersion uniformity, and stability. Findings from Transmission Electron Microscopy (TEM) and Dynamic Light Scattering (DLS) analysis, including size distribution curves and mean deviation, revealed that the zinc oxide nanosuspension exhibited a narrow size distribution and optimal morphology. The observed discrepancy between the TEM and DLS results can be attributed to the difference in the measurement techniques. TEM directly measures the particle size, whilst DLS assesses the hydrodynamic diameter, which includes the effects of mild aggregation and solvation in the solution. This aggregation could lead to a slight increase in the measured particle size in DLS, as the particles may form clusters in the suspension. However, despite this aggregation, the narrow size distribution and low polydispersity index (less than 0.5) observed in both nanosuspension samples suggest that the aggregation does not significantly affect the stability or dispersion of the nanoparticles in the suspension. The observed discrepancy between the Transmission Electron Microscopy (TEM) and Dynamic Light Scattering (DLS) results can be attributed to the differences in their measurement techniques. TEM directly measures the actual particle size, whilst DLS assesses the hydrodynamic diameter, which accounts for the effects of mild aggregation and solvation in the solution. This aggregation may result in a slight increase in the measured particle size during DLS analysis as particles can form clusters within the suspension. Despite the potential for aggregation, the narrow size distribution and low polydispersity index (less than 0.5) observed in both nanosuspension samples indicate that such aggregation does not significantly impact the stability or dispersion of the nanoparticles in the suspension. This suggests that the nanosuspensions maintain effective stability and uniformity, which are crucial for their intended applications. The polydispersity index (PDI), which is less than 0.5, indicates a uniform dispersion of nanoparticles in both nanosuspension samples and suggests a narrow particle size distribution (Gulsun et al. 2018). Additionally, the low *Z*‐average values (CaO = 199.2 nm, ZnO = 193.5 nm) and the small width of the size distribution curve for the ZnO nanosuspension further signify that this sample exhibits superior dispersion of nanoparticles within the suspension (Sarmphim et al. 2017). In this study, stability was assessed through zeta potential measurements, and our results suggest that both samples can be classified as stable nanosuspensions (Müller et al. 2001). However, the ZnO nanosuspension, due to its higher zeta potential value, can be considered more stable and exhibits greater absorbency than the CaO nanosuspension (Clogston and Patri 2011; Li et al. 2007). Additionally, long‐term zeta potential measurements further substantiate the superior stability of the ZnO nanosuspension. Over a 30‐day period, the ZnO sample maintained a consistently higher surface charge compared to the CaO sample, indicating sustained electrostatic repulsion and a reduced risk of particle aggregation. This prolonged stability is critical for ensuring uniform dispersion and maximising the antifungal efficacy of the nanosuspensions in practical applications (Honary and Zahir 2013; Danaei et al. 2018). These findings reinforce the suitability of ZnO nanosuspension as a robust and reliable candidate for future formulations.

The higher contact angle of CaO nanosuspension represents less solubility and higher surface attraction that might cause the formation of aggregation and these findings are consistent with the studies conducted by Karunakaran, Surya, et al. (2015). This reduced wettability may contribute to the weaker antifungal performance of CaO nanosuspension, as lower solubility and higher aggregation limit the bioavailability of active nanoparticles (Bhattacharya et al. 2019). In contrast, ZnO nanosuspension, with a lower contact angle and higher stability, exhibited significantly better antifungal activity against *N. dimidiatum*. In this study, the focus was primarily on identifying the most effective treatment against *Neoscytalidium dimidiatum*. The effect of concentration on the inhibitory activity will be evaluated in future studies to determine the optimal concentration for maximum efficacy. Studies on the preparation of azoxystrobin nanosuspension using the wet milling method have demonstrated that this formulation can reduce the mean diameter to 238.1 nm. This reduction in mean diameter enhances the antifungal activity and solubility of azoxystrobin (Yao et al. 2018). In the current study, we investigated the antifungal effects of various compounds on the mycelial growth of *Neoscytalidium dimidiatum*. This fungus is known by several synonyms, including *Torula dimidiata*, *Hendersonula toruloidea*, *Natrassia mangiferae*, *Scytalidium dimidiatum*, *Scytalidium hyalinum*, *Fusicoccum dimidiatum*, and *Neoscytalidium hyalinum*. Recent molecular studies have identified that four species within the Botryosphaeriaceae family—*N. dimidiatum*, *N. novaehollandiae* , and *N. orchidacearum*—are now considered synonyms of *N. dimidiatum* (Zhang et al. 2021). *N. dimidiatum* is recognised as a significant trunk pathogen affecting fruit, forest, and ornamental trees worldwide (Mayorquin et al. 2016; Nouri, Mohammadi, and Mirabolfathy 2018; Guney et al. 2022; Gusella et al. 2023). This fungal pathogen has also been reported to cause cankers and dieback in various tree species in Iran (Mirzaee et al. 2002; Nazerian et al. 2015; Yeganeh and Mohammadi 2021). Recently, it has been isolated and identified as a primary fungal trunk pathogen of pistachio trees in Iran (Sohrabi 2020) and in other countries (Dervis et al. 2019; Kurt et al. 2019). Our experimental results indicate that three of the eight treatments—peracetic acid, zinc oxide suspension, and copper oxychloride—completely inhibited the mycelial growth of *N. dimidiatum* in vitro. In contrast, polyethylene glycol, calcium oxide (both pure and as a nanosuspension), and pure zinc oxide failed to provide satisfactory control. Previous studies have shown that fungicides can have varying effects on the mycelial growth of *Botryosphaeriaceae* species, including *N. dimidiatum*. For instance, in a study focused on *N. dimidiatum*, commonly responsible for stem canker on Royal Poinciana, Cidely was top reported as the most effective fungicide against this pathogen (Al Raish et al. 2020). In similar research, fluazinam, thiophanate–methyl, tebuconazole, and boscalid + pyraclostrobin were reported as effective compounds in controlling the mycelium growth of this pathogen in vitro (Sakçı et al. 2022).

Although promising results were observed in the laboratory setting, scalability remains a key concern. The ability to produce nanosuspensions on a large scale whilst maintaining the consistency of particle size, stability, and efficacy presents challenges. Current methods used in this study, including wet milling, may be difficult to scale up economically or efficiently for field applications. Additionally, the long‐term stability of the nanosuspensions under field conditions—as well as temperature fluctuations, moisture, and UV exposure—has not been fully investigated. Further work is needed to assess the economic feasibility, environmental impact, and effectiveness of scaling up the production of zinc oxide and calcium oxide nanosuspensions for widespread agricultural use. Whilst nanosuspensions have demonstrated promising antifungal activity in vitro, additional research is required to translate these findings into practical field applications. Key factors such as formulation stability, delivery mechanisms, and environmental persistence must be optimised to ensure consistent efficacy under field conditions. Moreover, cost‐effectiveness and regulatory approval will be critical considerations for large‐scale agricultural implementation. Future studies should explore the integration of nanosuspensions into existing disease management programmes and assess their impact on beneficial microbial communities in the soil and plant microbiome.

Previous studies have shown that zinc oxide compounds exhibited considerable antimicrobial and antifungal activity (Singh and Nanda 2013). The antifungal activity of ZnO nanoparticles is primarily due to their ability to generate ROS, which leads to oxidative stress, protein dysfunction, and DNA damage in fungal cells (Raghupathi et al. 2011; Dimkpa et al. 2012). Additionally, ZnO interacts with fungal membranes through electrostatic attraction, causing increased permeability and leakage of intracellular components (Lallo da Silva et al. 2019). On the other hand, CaO nanoparticles act by altering the pH of the fungal microenvironment and disrupting metabolic processes, though their limited antifungal activity in this study suggests that this mechanism is less effective compared to ZnO‐mediated oxidative stress (Kumar et al. 2019). Furthermore, compounds derived from zinc oxide are considered low‐cost, biocompatible, and environmentally friendly (Mandal et al. 2022). The fungicidal effects of zinc oxide are particularly evident in studies such as the one involving ZnO‐encapsulated essential oil from Zataria multiflora, which has shown effectiveness against Alternaria solani (Akhtari et al. 2022). Copper oxychloride, also known as dicopper chloride trihydroxide, is classified within the M FRAC Group and is recommended for use against various fungal, bacterial, and Oomycete plant diseases, including citrus gummosis, potato late blight, walnut anthracnose, cucumber angular leaf spot, and citrus blight caused by *N. dimidiatum* (Sheikhi et al. 2017). Both pure and suspension concentrates of calcium oxide yielded similar results, demonstrating a reduction in the mycelial growth of *N. dimidiatum*; however, their effectiveness was significantly lower compared to that of the zinc oxide suspension and peracetic acid (DSP). Amongst the treatments examined, PEG 400 and peracetic acid were used as adjuvants. Notably, peracetic acid exhibited a substantial impact on the mycelial growth rate of *N. dimidiatum*, whilst PEG 400 showed no effect on this pathogen. Peracetic acid can be regarded as a strong synergist when combined with zinc oxide, although it does not exhibit the same synergy with calcium oxide.

The underlying mechanism of this synergy may involve reactive oxygen species (ROS) generation and membrane disruption. ZnO nanoparticles are known to produce hydroxyl radicals (•OH) and superoxide anions (O_2_^−^) upon interaction with fungal cells, leading to oxidative stress and cell damage (Lallo da Silva et al. 2019; Raghupathi et al. 2011). PAA enhances this effect by disrupting fungal cell membranes (Kitis 2004) and increasing ZnO nanoparticle penetration, thereby amplifying oxidative damage and improving antifungal efficacy (Dimkpa et al. 2012). Peracetic acid, also known as peroxyacetic acid (PAA), is a highly effective disinfectant that targets a wide range of microorganisms, including various bacteria and viruses (Kitis 2004; Bernet et al. 2005). Synergistic effects can be observed when PAA is combined with different compounds, such as fungicides and organic sanitizers. For instance, Ayoub et al. (2018) reported a synergistic action between SWITCH (produced by Syngenta, Switzerland) and the organic sanitizer PERACLEAN (PAA)5 from Evonik Industries, Germany, for controlling Botrytis cinerea. Previous research has established that PAA alone possesses significant antifungal properties. Mari et al. (2004) demonstrated its antifungal activity against Monilinia laxa and Rhizopus stolonifer. Additionally, Stefanello et al. (2020) reported that PAA exhibits high antifungal efficacy against various moulds, including species of Aspergillus and Paecilomyces. These findings support the potential of peracetic acid as a powerful agent in the management of fungal pathogens. Similarly, in a separate study, Yang et al. (2022) demonstrated that peracetic acid (PAA) can significantly reduce the presence of fungi responsible for black stains in Asian pears. These findings position PAA as a promising alternative for controlling certain fungal species and plant diseases. Our research found that the advanced nanosuspension formulation of zinc oxide enhanced its antifungal efficacy against *N. dimidiatum*. Nanosuspension technology addresses the challenges associated with poorly water‐soluble compounds, thereby increasing their bioavailability and activity. In essence, key characteristics of antifungal compounds, such as stability and effectiveness, can be significantly improved through the application of nanosuspension technology. Our results align with previous studies on antifungal agents, which have indicated that nanosuspension formulations are not only technically simpler and more cost‐effective but also yield physically stable products compared to other formulations (Siekmann and Westesen 1995; Müller and Peters 1998). The findings from this study revealed that two *N. dimidiatum* isolates exhibited varying degrees of sensitivity to the treatments, particularly the pure powder of zinc oxide. Specifically, the pure zinc oxide was more effective against the mycelial growth of the IRNM235 isolate, whilst the IRNM237 isolate showed greater resistance to this treatment. Our results corroborate those of James et al. (2017), who investigated the effects of various antifungal agents on *N. dimidiatum* isolates obtained from clinical specimens. This pattern is also consistent with the results of fungicide evaluations against other Botryosphaeriaceae species, such as Diplodia and Neofusicoccum, associated with Botryosphaeria canker in grapevines in Chile (Torres et al. 2013). Deising et al. (2008) noted that fungicide resistance may arise from genetic factors, including gene mutations and encoding in fungal pathogens. Consequently, different isolates of fungal pathogens may exhibit varied responses to fungicides. The unique properties of zinc oxide nanoparticles, such as their solubility in water and enhanced efficacy of active ingredients, highlight their promising potential in managing *N. dimidiatum*. However, further research is warranted to explore the feasibility of loading conventional inorganic compounds onto different isolates of *N. dimidiatum*.

In the present study, we propose an industrial method for synthesising stable nanosuspensions of zinc and calcium oxide, achieving a mean diameter of less than 10 nm. We investigated the fungicidal effects of these synthesised nanosuspensions, along with two additive materials, in comparison to pure calcium and zinc oxide on the mycelial growth rate of *N. dimidiatum*. This study marks the inaugural effort to synthesise and evaluate zinc oxide (ZnO) and calcium oxide (CaO) nanosuspensions for the control of *N. dimidiatum*. Utilising a top‐down milling approach, we successfully developed a stable nanosuspension that exhibits improved solubility and bioavailability. Unlike conventional fungicide formulations, these nanosuspensions provide a targeted, eco‐friendly alternative, significantly reducing environmental impact. These findings pave the way for the application of nanotechnology in sustainable plant disease management. According to the in vitro results, zinc oxide nanosuspension completely inhibited the mycelial growth of *N. dimidiatum*. Additionally, the experiments demonstrated that peracetic acid exhibited synergistic effects with zinc oxide (wp) on the mycelial growth rate of *N. dimidiatum*. Further research into the potential application of zinc oxide nanosuspension and peracetic acid under orchard conditions would be beneficial.

## Author Contributions

**Seyedeh Fatemeh Shojaei:** investigation, writing – original draft, conceptualization, formal analysis, funding acquisition, software, methodology. **Hamid Mohammadi:** supervision, validation, writing – review and editing, resources, data curation. **Nazanin Foroutan:** writing – review and editing, methodology, resources. **Saleh Panahandeh:** conceptualization, methodology, investigation, project administration, software, visualization.

## Conflicts of Interest

The authors declare no conflicts of interest.

## Data Availability

Data sharing is not applicable to this article as no new data were created or analyzed in this study.
